# Depot-Specific Adipose Tissue Metabolite Profiles and Corresponding Changes Following Aerobic Exercise

**DOI:** 10.3389/fendo.2018.00759

**Published:** 2018-12-11

**Authors:** Andrea M. Brennan, Andre Tchernof, Robert E. Gerszten, Theresa E. Cowan, Robert Ross

**Affiliations:** ^1^School of Kinesiology and Health Studies, Queen's University, Kingston, ON, Canada; ^2^Endocrinology and Nephrology, University Hospital of Quebec, Quebec, QC, Canada; ^3^School of Nutrition, Laval University, Quebec, QC, Canada; ^4^Quebec Heart and Lung Institute, Quebec, QC, Canada; ^5^Division of Cardiovascular Medicine, Beth Israel Deaconess Medical Center, Boston, MA, United States

**Keywords:** adipose tissue, metabolomics, aerobic exercise, body composition, metabolism

## Abstract

**Objectives:** Total, visceral, and abdominal subcutaneous adipose tissue (AT) depots have distinct associations with cardiometabolic health; however, the metabolite profiles that characterize each AT depot and its reduction following exercise are poorly understood. Our objectives were to (1) assess the independent associations between identified metabolites and total, visceral and abdominal subcutaneous AT; and (2) examine whether changes in metabolite concentrations and AT mass following aerobic exercise are associated.

**Methods:** A secondary analysis was performed in 103 middle-aged abdominally obese men and women {[mean (SD)], 52.4 (8.0) years} randomized into one of four groups varying in exercise amount and intensity for 6 months duration: high amount high intensity, high amount low intensity, low amount low intensity, and control. One hundred and forty seven metabolites were profiled by liquid chromatography-tandem mass spectrometry. AT mass was measured by magnetic resonance imaging (MRI).

**Results:** Individual metabolite associations with AT depots confirmed several established cross-sectional relationships between the obesity phenotype and metabolic pathways. Collapsed across exercise groups, reduction in visceral AT predicted increases in pyroglutamic acid (B = −0.41) and TCA cycle intermediates [succinic (B = −0.41) and fumaric acid (B = −0.20)], independent of change in total AT. Changes in UDP-GlcNAc (B = 0.43), pyroglutamic acid (B = −0.35), histidine (B = 0.20), citric acid/isocitric acid (B = −0.20), and creatine (B = 0.27) were significantly associated with changes in total AT (false discovery rate = 0.1).

**Conclusions:** Our findings point to potential biomarkers of depot-specific AT reduction that may play a direct role in mediating cardiometabolic improvements.

## Introduction

Excess accumulation of adiposity, in particular abdominal adipose tissue (AT), is associated with morbidity and mortality, independent of age and sex (1). To explain underlying metabolic alterations in response to AT accumulation that may result in subsequent health decline, current observations focus on a small number of measured biomarkers including lipoproteins, insulin, and glucose (2). Metabolomics, the assessment of large numbers of small-molecule metabolites, affords the opportunity to gain a broader understanding of the activity of metabolic pathways affected by excess adiposity (3). The substrates and products of metabolism under conditions of excess adiposity may serve as biomarkers for AT dysfunction or directly mediate AT pathology as initiators or regulatory signals of disease processes.

Total, visceral and abdominal subcutaneous AT depots have distinct associations with cardiometabolic health (4); however, the metabolite profile that characterizes each AT depot and mediates risk for disease is not understood. Magnetic resonance imaging (MRI), combined with metabolomics technologies, allows for exploitation of biomarkers and/or molecular mechanisms that both characterize the phenotype associated with specific AT depot accumulation and explain the impact of AT reduction on circulating metabolites and corresponding improvement in cardiometabolic risk. Current cross-sectional observations relating specific AT depots and the metabolome are limited and inconsistent. Boulet et al. observed positive associations between the branched chain amino acids (BCAAs), alanine, tyrosine, and glutamate with both subcutaneous and visceral AT (5), while Schlecht et al. did not identify any significant associations between visceral and abdominal subcutaneous AT and 20 serum metabolites measured (6). To our knowledge, no prior observations exist to describe corresponding changes of metabolites and AT depots following exposure to physiologic perturbations including exercise.

We previously conducted and published primary findings from a randomized controlled trial examining the separate effects of exercise amount and/or intensity on abdominal obesity and glucose tolerance (7, 8). In this secondary analysis, using MRI-derived AT depot data in addition to plasma metabolite profiles collected before and after the exercise intervention we aim to (1) assess the independent associations between identified metabolites and total, visceral, and abdominal subcutaneous AT; and (2) examine whether metabolite concentrations and AT mass change concurrently following chronic aerobic exercise. Our observations may help to identify novel biomarkers of both excess total and regional AT and efficacy of treatment.

## Materials and Methods

### Participants

Details of the study design and methods have been published previously (7) as are the findings from the primary analysis (8). Briefly, we conducted a 24 weeks, single center, randomized controlled trial with a parallel group design between September 2009 and May 2013. Three hundred abdominally obese adults were randomized into one of four treatments: control (no exercise), low amount low intensity (LALI; 180 and 300 kcal/session for women and men, respectively, at 50% VO_2_peak), high amount low intensity (HALI; 360 and 600 kcal/session, respectively, at 50% VO_2_peak), and high amount high intensity (HAHI; 360 and 600 kcal/session, respectively, at 75% VO_2_peak). Exercise sessions were supervised and performed 5 days/week for the duration of the trial. All participants provided informed consent prior to participation and the protocols used in the original investigation and this secondary analysis were approved by the Queen's University Health Sciences Research Ethics Board.

Participants from the original trial were excluded if they did not have both pre- and post- MRI data (*n* = 171) and if there were issues with the MRI protocol (image quality and positioning pre- and/or post-treatment) (*n* = 23) which resulted in a study sample of 103 participants: Control, *n* = 20; LALI, *n* = 24; HALI, *n* = 30; HAHI, *n* = 29. Participant characteristics in Table 1 do not replicate prior analyses derived from the primary trial.

**Table 1:** Participant characteristics at baseline and 24 weeks.

**Characteristics**	**Control**		**LALI**		**HALI**		**HAHI**	
	**Baseline**	**24 weeks**	**Baseline**	**24 weeks**	**Baseline**	**24 weeks**	**Baseline**	**24 weeks**
Age, *years*	55.1 ± 6.6		52.5 ± 8.0		51.8 ± 8.3		52.8 ± 7.4
Sex F:M	10:10		14:10		20:11		19:11
**ANTHROPOMETRIC**								
Weight, *kg*	89.7 ± 17.6	89.0 ± 17.9	94.6 ± 14.6	90.1 ± 14.2*	92.9 ± 14.9	86.7 ± 14.9*	95.2 ± 14.3	88.9 ± 12.5*
WC, *cm*	107.0 ± 11.2	105.8 ± 11.6	111.9 ± 10.7	106.8 ± 10.3*	108.8 ± 9.4	102.2 ± 9.9*	109.4 ± 11.8	103.2 ± 10.5*
BMI, *kg/m^2^*	30.5 ± 3.6	30.4 ± 3.8	33.2 ± 4.3	31.6 ± 4.5*	32.6 ± 4.1	30.4 ± 4.3*	32.5 ± 3.7	30.4 ± 3.2*
**ADIPOSE TISSUE MASS**								
Total AT, *kg*	37.0 ± 8.3	37.2 ± 9.2	43.0 ± 8.8	39.7 ± 9.9*	41.5 ± 8.1	37.2 ± 9.1*	43.0 ± 8.4	37.9 ± 7.6*
Total SAT, *kg*	28.1 ± 7.2	28.3 ± 7.9	34.0 ± 8.2	31.9 ± 9.4*	32.8 ± 8.0	29.6 ± 8.4*	33.8 ± 7.5	30.0 ± 6.9*
Total abdominal AT, *kg*	8.7 ± 2.2	8.6 ± 2.4	9.8 ± 2.0	8.8 ± 2.2*	9.3 ± 2.0	8.2 ± 2.1*	9.7 ± 2.7	8.3 ± 2.2*
VAT, *kg*	3.3 ± 1.5	3.2 ± 1.5	3.2 ± 1.3	2.6 ± 1.0*	3.0 ± 1.3	2.5 ± 1.2*	3.2 ± 1.5	2.6 ± 1.2*
ASAT, *kg*	5.1 ± 1.5	5.1 ± 1.6	6.3 ± 1.5	5.9 ± 1.8*	6.0 ± 1.6	5.4 ± 1.7*	6.1 ± 1.7	5.3 ± 1.4*
**METABOLIC**								
HOMA-IR	2.7 ± 1.5	2.5 ± 1.2	2.4 ± 1.9	1.9 ± 1.2*	2.0 ± 1.3	1.6 ± 1.2*	2.3 ± 1.3	1.6 ± 0.9*

* *Significantly different from controls (p < 0.01). Values presented are mean ± SD. F, female; m, male; WC, waist circumference; BMI, body mass index; AT, adipose tissue; SAT, subcutaneous adipose tissue; VAT, visceral adipose tissue; ASAT, abdominal subcutaneous adipose tissue; LALI, low amount low intensity; HALI, high amount low intensity; HAHI, high amount high intensity*.

### Assessment of Body Composition

Whole body AT was measured by MRI at baseline and following treatment by a 1.5 Tesla magnet at Kingston General Hospital using a protocol previously described (9, 10). Briefly, participants entered the magnet in a prone position, feet first and the L4-L5 intervertebral space was landmarked using a sagittal scout image. 10 mm thick images, 40 mm apart were taken from L4-L5 to the lower extremities (feet). The participants were then required to exit the magnet and re-enter head first with their arms extended. The L4-L5 inter-vertebral space was relocated and 10 mm thick images 40 mm apart were taken from L4-L5 to the upper extremities (hands). Specialized image analysis software (sliceOmatic version 5.0, TomoVision, Montreal, QC) was used to quantify the tissue depots. Total AT was derived using all images (~41 images per participant). Abdominal subcutaneous and visceral AT were determined using 5 consecutive images beginning one image below L4-L5, L4-L5 and three images above.

Due to scheduling issues, MRI scans for 22 participants were obtained more than 2 weeks after the last exercise session or the end of the 6 months monitoring period (Control, *n* = 6; LALI, *n* = 4; HALI, *n* = 8; HAHI, *n* = 4). For these cases, total weight derived from the MRI analysis was compared to the anthropometric weight taken at week 24. The average weight difference for these cases was 1.8 kg (±1.8). Exclusion of these participants from the analysis revealed no significant difference in our overall findings, thus, they were included in our final sample.

### Plasma Sampling and Metabolite Profiling

Fasting plasma samples were acquired via the antecubital vein at baseline prior to an OGTT and again at least 48 h after the last exercise session at 24 weeks. The plasma was immediately separated by centrifugation (10 min at 4,250 rpm) and stored at −80°C.

Targeted metabolomic profiling of plasma samples using LC-MS/MS in positive and negative electrospray ionization modes was performed and is described elsewhere (11). Briefly, in positive mode, normal phase hydrophilic interaction chromatography (HILIC) using a 2.1 × 150 mm 3 μm Atlantis column (Waters) was coupled to a 4,000 QTrap triple quadrupole mass spectrometer (Applied Biosystems/Sciex) equipped with an electrospray ionization source for targeted detection of 139 metabolites using a dynamic multiple reaction monitoring (dMRM) mechanism. In negative mode, HILIC chromatography using a 2.1 × 100mm 3.5 μm Xbridge Amide column (Waters) was coupled to an Agilent 6,490 triple quadrupole mass spectrometer equipped with an electrospray ionization source for targeted detection of 70 metabolites using dMRM. Metabolite peaks were integrated using Sciex MultiQuant software (positive mode) or Agilent Masshunter Quantitative software (negative mode). All metabolite peaks were manually reviewed for peak quality in a blinded manner.

### Statistical Analysis

All metabolites were log-transformed to approximate a normal distribution and to reduce heteroscedasticity and analyses were adjusted for age and sex. Principal component analysis (PCA) was used to reduce dimensionality of the metabolite data set at baseline. Nineteen main principal factors were derived after orthogonal Varimax rotation (Table 2). Factors were retained if they had an eigenvalue >1 and individual metabolites with a factor load >|0.4| for a given PCA-derived factor were included. Scores were calculated for each participant based on standardized scoring coefficients. Cross-sectional associations between PCA factor scores and AT depots (total, abdominal, abdominal subcutaneous, visceral AT) were assessed with multiple linear regression. In addition, multiple linear regression was used to determine cross-sectional associations between individual metabolites and AT depots.

**Table 2:** Principal Component Analysis Factors.

**Factors**	**Metabolites**	**Eigenvalue**	**Variance, %**
1	Lysine, glutamine, histidine, methionine, ornithine, arginine, NMMA, asparagine, tyrosine, proline, xanthurenate, alanine, aspartate cysteine, threonine, valine, citrulline, leucine, tryptophan, isoleucine, ADMA/SDMA	21.002	14.385
2	C10-carnitine, C8-carnitine, C12-carnitine, C6-carnitine, C14-carnitine, C7-carnitine, C5-glutarycarnitine, C9-carnitine, C16-carnitine	10.258	7.026
3	Lactic acid, hypoxanthine, pyruvic acid, inosine, alpha-ketoglutaric acid, 2-ketoisovaleric acid, ketoisocaproic acid, xanthosine,	9.276	6.354
4	2-aminoadipic acid, 2-hydroxyglutaric acid, malic acid, phosphocreatine, quinolinic acid, D-Gluconic acid, oxalic acid, citric acid/isocitric acid, malonic acid	7.653	5.242
5	Kynurenine, anthranilic acid, indole-3-carboxylic acid,	6.477	4.436
6	C18:2-carnitine, C18:1 carnitine, C18-carnitine, C16-carnitine	5.672	3.885
7	Uridine, uracil, xanthine	4.942	3.385
8	C4-butyryl carnitine, C3-carnitine, C5-valerylcarnitine, carnitine	4.838	3.313
9	Glycocholic acid, taurodeoxycholic acid, taurocholic acid, glycochenodeoxycholic acid	4.098	3.824
10	Xanthurenic acid, indole-3-lactic acid, kynurenic acid	3.824	2.620
11	Glucose/fructose/galactose, 2-deoxyuridine, uric acid	3.318	2.272
12	ADP, AMP, ATP	3.126	2.141
13	3-aminoisobutyric acid	2.937	2.012
14	Taurine	2.862	1.960
15	Allantoin	2.649	1.814
16	Thiamine, pantothenic acid	2.443	1.674
17	UDP-GlcNAc, UDP-glucose-galactose	2.315	1.585
18	Trimethylamine-N-oxide, cysteamine	2.066	1.415
19	5-adenosyl-homocysteine, glycine	1.924	1.318

*Factors were retained if they had an eigenvalue >1. Only those metabolites with a factor load >|0.4| for a given PCA-derived factor are shown*.

To determine the association between changes in measures of adiposity and change in metabolite concentrations, partial least squares regression (PLS) was first used to reduce the number of metabolites based on their predictive performance for the outcome (AT depot). The Variable of Importance in the Projection Statistic (VIP) was calculated and used to determine the relative contribution of each metabolite. Metabolites with VIP scores ≥1 were extracted for further interrogation. Multiple linear regression was used to determine associations between the changes in metabolites extracted by PLS and AT reduction, adjusted for baseline metabolite concentration. Analyses were then adjusted for total AT. In all of the above analyses, *p*-values were FDR-adjusted (*q*-value = 10%) for multiple testing using the Benjamini-Hochberg method.

Selected metabolites whose change was associated with change in the AT depots at *p* < 0.05 were further interrogated through multiple linear regression to determine combined and independent associations with measures of glucose and insulin metabolism (HOMA-IR, 2 h glucose, insulin AUC). Statistical significance was evaluated at *p* < 0.05. All analyses were performed using SPSS Statistics (Version 24).

## Results

Participant characteristics before and after treatment are summarized in Table 1. Significant reductions in all AT depots, HOMA-IR, and insulin AUC were observed in each exercise group compared to controls; however, there were no differences between exercise groups (*p* < 0.05). Thus, subsequent analyses are collapsed across exercise group.

PCA was used to group highly correlated metabolites into fewer factors (Table 2). Total, abdominal, and abdominal subcutaneous AT were positively associated with Factor 3 (purine and BCAA metabolites), Factor 5 (tryptophan metabolites), and Factor 15 (allantoin) (*p* < 0.05). Visceral AT was positively associated with Factor 5 (*p* < 0.05). Individual metabolite associations with AT depots confirmed several established cross-sectional relationships between the obesity phenotype and metabolic pathways as illustrated in Table 3 (all FDR < 0.10). After adjusting for total AT, kynurenic acid (B = 0.18, *p* = 2.9 × 10^−5^), tyrosine (B = 0.09, *p* = 0.049), valine (B = 0.10, *p* = 0.033), and homogentisic acid (B = 0.10, *p* = 0.018) were significantly associated with abdominal AT. Similarly, visceral AT was significantly associated with phenylalanine (B = 0.20, *p* = 0.003), kynurenic acid (B = 0.19, *p* = 0.003), homogentisic acid (B = 0.14, *p* = 0.015), and the BCAAs (B = 0.13–0.20, *p* = 0.003–0.041) after adjusting for total AT.

**Table 3:** Cross-sectional associations between metabolites and AT depots.

**AT depot**	**Metabolite**	**Std. ß**	***P*-Value**	**Adjusted for Total AT**	
				**Std. ß**	***P*-value**
Total AT	Anthranilic acid	0.39	4.0 × 10^−5^	
	Kynurenine	0.39	4.4 × 10^−5^	
	Proline	0.35	4.0 × 10^−4^	
	Uric acid	0.35	0.002	
	Tyrosine	0.31	0.002	
	Allantoin	0.31	0.003	
	Quinolinic acid	0.29	0.004	
	Phenylalanine	0.31	0.005	
	1,5-AG-1-deoxyglucose	0.27	0.006	
	Hydroxyphenylpyruvic acid	0.27	0.007	
	N-Acetyl-L Methionine	0.27	0.007	
	Kynurenic acid	0.26	0.009	
	Pseudouridine	0.26	0.010	
Abdominal AT	Anthranilic acid	0.39	2.1 × 10^−5^		NS
	Kynurenine	0.38	3.7 × 10^−5^		NS
	Proline	0.35	1.7 × 10^−4^		NS
	Kynurenic acid	0.36	2.0 × 10^−4^	0.18	2.9 × 10^−5^
	Uric acid	0.39	2.6 × 10^−4^		NS
	Tyrosine	0.35	3.0 × 10^−4^	0.09	0.049
	Phenylalanine	0.37	3.6 × 10^−4^		NS
	Isoleucine	0.35	0.002		NS
	Allantoin	0.29	0.004		NS
	Cis-trans-hydroxyproline	0.27	0.005		NS
	Valine	0.28	0.007	0.10	0.033
	2-ketoisovaleric acid	0.26	0.007		NS
	Xanthine	0.25	0.008		NS
	Quinolinic acid	0.25	0.009		NS
	Carnitine	0.26	0.009		NS
	Homogentisic acid	0.24	0.011	0.10	0.018
	N-Acetyl-L-Methionine	0.24	0.011		NS
	Oxaloacetic acid	0.24	0.012		NS
	Alanine	0.23	0.014		NS
	1,5-AG-1-deoxyglucose	0.23	0.014		NS
Abdominal Subcutaneous AT	Anthranilic acid	0.39	3.0 × 10^−5^		NS
	Kynurenine	0.37	9.3 × 10^−5^		NS
	Proline	0.33	0.001		NS
	Uric acid	0.37	0.001		NS
	Kynurenic acid	0.31	0.002		NS
	Allantoin	0.31	0.002		NS
	Tyrosine	0.30	0.002		NS
	Cis-trans-hydroxyproline	0.29	0.004		NS
	Hydroxyphenylpyruvic acid	0.28	0.004		NS
	Carnitine	0.28	0.005		NS
Visceral AT	Phenylalanine	0.30	6.0 × 10^−5^	0.20	0.003
	Kynurenic acid	0.26	2.4 × 10^−4^	0.19	0.003
	Isoleucine	0.29	3.7 × 10^−4^	0.20	0.003
	Tyrosine	0.22	0.002		NS
	Homogentisic acid	0.21	0.003	0.14	0.015
	Leucine	0.23	0.005	0.18	0.009
	Valine	0.21	0.005	0.13	0.041

*Only significant metabolites using FDR-adjusted p-value are listed. NS, not significant; AT, adipose tissue. All analyses adjusted for age and sex*.

**Figure 1:**
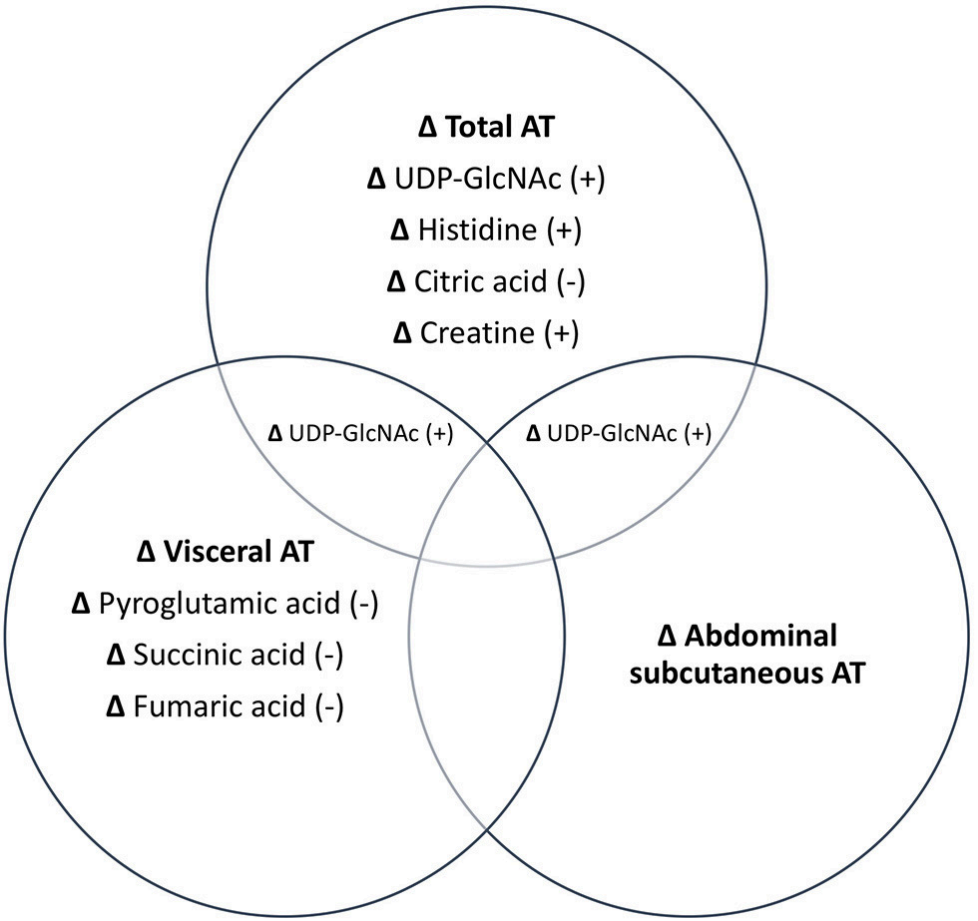
Independent and combined associations between changes in metabolites and changes in AT depots. All metabolites listed are significant at *p* < 0.05. +, positive association; –, negative association.

Associations between changes in AT depots and changes in metabolites are illustrated in Figure 1. PLS analyses reduced the number of examined metabolites to 49, 47, 48, and 45 for total, abdominal, abdominal subcutaneous, and visceral AT, respectively based on VIP scores ≥1. After FDR-adjustment, changes in UDP-GlcNAc (B = 0.43), pyroglutamic acid (B = −0.35), histidine (B = 0.20), citric acid/isocitric acid (B = −0.20), and creatine (B = 0.27) were significantly associated with changes in total AT. Changes in UDP-GlcNAc (B = 0.38) and pyroglutamic acid (B = −0.39) were significantly associated with change in abdominal AT. Only change in UDP-GlcNAc (B = 0.35) was significantly associated with change in abdominal subcutaneous AT. Change in pyroglutamic acid (B = −0.44), succinic acid (B = −0.42), fumaric acid (B = −0.29), and UDP-GlcNAc (B = 0.28) were significantly associated with visceral AT changes. Pyroglutamic acid (B = −0.39) remained significantly associated with change in abdominal AT after adjusting for change in total AT (*p* < 0.05). Pyroglutamic acid (B = −0.41), succinic acid (B = −0.41), and fumaric acid (B = −0.20), remained significantly associated with visceral AT after adjusting for total AT (*p* < 0.05).

Metabolites whose changes were significantly associated with changes in AT depots were interrogated further to determine combined and independent associations with changes in glucose and insulin metabolism (HOMA-IR, 2 h glucose and insulin AUC), shown in Table 4. Univariate associations revealed that change in C5-glutarylcarnitine (B = 0.22) and inosine (B = −0.32) were significantly associated with change in 2 h glucose; change in creatine (B = 0.24) and UDP-GlcNAc (B = 0.25) were significantly associated with change in insulin AUC; and change in C8-carnitine, UDP-GlcNAc (B = 0.26), glutamate (B = 0.20), and succinic acid (B = −0.25) were significantly associated with change in HOMA-IR (B = −0.30) (all *p* < 0.05). After adjusting for its related AT depot change, C5-glutarylcarnitine and inosine remained significantly associated with change in 2 h glucose (*p* < 0.05); change in creatine remained significantly associated with change in insulin AUC; and change in C8-carnitine, remained significantly associated with change in HOMA-IR. UDP-GlcNAc, glutamate, and succinic acid were no longer associated with measures of insulin and glucose metabolism after adjusting for adiposity (*p* > 0.05).

**Table 4:** Independent associations between changes in metabolites, adiposity measures, and measures of glucose and insulin metabolism.

**Glucose or insulin measure**	**Metabolite**	**Std.ß**
2-hour glucose	C5-glutarylcarnitine^§^ Inosine^§^	0.22 −0.32
Insulin AUC	Creatine^§^ UDP-GlcNAc	0.24 0.25
HOMA-IR	C8-carnitine^§^ UDP-GlcNAc Glutamate Succinic Acid	−0.28 0.26 0.20 −0.25

§ *After adjusting for changes in adiposity, metabolites remained significantly associated with measures of glucose and insulin metabolism at p < 0.05. All analyses adjusted for age, sex and baseline glucose/insulin measure*.

## Discussion

To our knowledge, this is the first study to systematically explore the impact of depot-specific AT reduction following aerobic exercise on the plasma metabolome. Our primary finding was that reduction in visceral AT independently predicted increases in pyroglutamic acid and TCA cycle intermediates (succinic and fumaric acid) in men and women with abdominal obesity. Though preliminary, our observations point to novel biomarkers of total and regional AT reduction following exercise that may also mediate changes in cardiometabolic health. Given that routine measurement of visceral AT by MRI is not feasible in clinical practice, the discovery of blood-based biomarkers that can act as surrogate measures of visceral AT may improve excess visceral AT mass diagnosis in addition to improvements following treatment.

The combination of metabolomics and MRI affords the opportunity to extend the work of others to examine subtle variations in the metabolite network that are influenced by fluctuation in total and regional adiposity. We observed that visceral AT reduction was associated with increases in the TCA cycle intermediates, succinic acid and fumaric acid, independent of changes in total adiposity. While literature examining plasma TCA cycle intermediates and AT depots is scarce, findings from biopsy samples of adipocytes demonstrate lower mitochondrial citrate synthase and oxygen consumption rates in visceral adipocytes from obese compared to non-obese subjects (12). Combined with our findings, it is possible that visceral AT reduction following exercise helps to mediate the metabolic efficiency of the mitochondrial TCA cycle and is reflected by higher levels of TCA cycle intermediates in the plasma, though this finding is preliminary and requires confirmation with mechanistic studies (12). We also observed increases in pyroglutamic acid corresponding to reduced visceral AT. While its role is poorly understood, pyroglutamic acid is partly produced enzymatically from glutamate and may act as a glutamate reservoir (13). Boulet et al. previously observed increased glutamate concentrations with excess visceral AT (5). Thus, combined with our findings, it is possible that increased production of pyroglutamic acid via reductions in visceral adiposity attenuates circulating glutamate levels. We did not observe any significant metabolite changes that were independently associated with abdominal subcutaneous AT reduction, rather metabolite associations with this depot instead reflected changes in total adiposity.

Several cross-sectional observations exist that characterize the metabolic profile of individuals with obesity compared to their lean counterparts (14). Increased circulating levels of BCAAs, tryptophan (15, 16), tyrosine (15–18) and nucleotide metabolites (18) are consistently shown to be increased in those with obesity (15, 17–22). Few observations exist that evaluate AT depot-specific metabolite profiles, especially after adjusting for total adiposity. Boulet et al. identified glutamate as the strongest independent predictor of visceral AT, while valine was independently associated with subcutaneous AT after adjustment for total AT (5). Here, we confirm prior metabolite associations with total adiposity and extend those observations by uncovering significant relations between higher visceral AT mass and corresponding circulating concentrations of BCAAs, independent of total AT.

It is well-established that excess adiposity, in particular visceral AT, is associated with cardiometabolic derangements (1); however, the underlying molecular mechanisms that explain the distinct contribution of each AT depot to risk are unclear. We observed that changes in UDP-GlcNAc (decrease), glutamate (decrease), and succinic acid (increase) predicted improvement in measures of glucose and insulin homeostasis, but not after adjusting for AT reduction. This suggests that part of the association between metabolic changes and cardiometabolic risk can be explained by changes in adiposity. In particular, we previously reported significant associations between UDP-GlcNAc and HOMA-IR (11). UDP-GlcNAc is a nucleotide sugar that is sensitive to changes in energy balance (23). It is a product of the hexosamine biosynthetic pathway (HBP) and a substrate for the O-linked N-acetylglucosamine transferase enzyme (OGT) that catalyzes protein modification through the addition of O-GlcNAc. Under conditions of chronic positive energy balance, the activity of the HBP and corresponding production of UDP-GlcNAc increases, along with OGT-catalyzed O-GlcNAcylation. Excessive O-GlcNAcylation directly interferes with insulin signaling pathways, resulting in reduced insulin sensitivity (23). Thus, combined with our present observations, part of the associated improvement in insulin resistance with UDP-GlcNAc reduction may be explained by changes in total AT.

A limitation of our study is that on average, few metabolites significantly changed with exercise compared to controls (11); thus, we cannot attribute the AT-metabolite associations to exercise-induced AT reduction, *per se*. It is unknown whether alternative perturbations including diet would elicit similar responses and it is possible that plasma metabolite changes would reflect the separate and combined influences of dietary and adiposity changes. Our findings are still relevant for addressing important gaps in the current literature regarding metabolic consequences of total and regional adiposity changes, regardless of the source of AT change. Additionally, our sample was relatively homogeneous which may have attenuated the strength of the observed relationships and limits the generalizability of our findings. However, we recently observed substantial interindividual variability in AT changes with exercise (24), therefore the range of AT loss varies across individuals.

In summary, our findings point to potential biomarkers of depot-specific AT accumulation and reduction that may play a direct role in mediating cardiometabolic improvements. These observations prompt future work for mechanistic studies to elucidate cause-and-effect relationships.

## Ethics Statement

This study was carried out in accordance with the recommendations of Health Sciences Research Ethics Board at Queen's University with written informed consent from all subjects. All subjects gave written informed consent in accordance with the Declaration of Helsinki. The protocol was approved by the Queen's University Health Sciences Research Ethics Board.

## Author Contributions

AB and RR conceived and designed the study. TC and AB analyzed the MRI images. RG contributed to metabolomics analysis of plasma samples. AB completed all statistical analyses. AB and RR drafted the manuscript. All authors edited and revised the manuscript with critical feedback given.

### Conflict of Interest Statement

The authors declare that the research was conducted in the absence of any commercial or financial relationships that could be construed as a potential conflict of interest.
